# Quantifying the performance of MEG source reconstruction using resting state data

**DOI:** 10.1016/j.neuroimage.2018.07.030

**Published:** 2018-11-01

**Authors:** Simon Little, James Bonaiuto, Sofie S. Meyer, Jose Lopez, Sven Bestmann, Gareth Barnes

**Affiliations:** a Department of Clinical and Movement Neurosciences, UCL Institute of Neurology, Queen Square, London, UK; b Centre de Neuroscience Cognitive, CNRS UMR 5229-Université Claude Bernard Lyon I, 69675, Bron Cedex, France; c Wellcome Centre for Human Neuroimaging, UCL Institute of Neurology, 12 Queen Square, London, UK; d Institute of Cognitive Neuroscience, University College London, London, WC1N 3AR, UK; e Institute of Neurology, University College London, London, WC1N 1PJ, UK; f Electronic Engineering Department, Universidad de Antioquia, UdeA, Calle 70 No. 52-21, Medellín, Colombia

**Keywords:** Magnetoencephalography, Resting state, Hidden Markov model, Head-cast, Inversion, Empirical Bayesian beamformer, Minimum norm, LORETA, Multiple sparse priors, Forward model, Resolution

## Abstract

In magnetoencephalography (MEG) research there are a variety of inversion methods to transform sensor data into estimates of brain activity. Each new inversion scheme is generally justified against a specific simulated or task scenario. The choice of this scenario will however have a large impact on how well the scheme performs. We describe a method with minimal selection bias to quantify algorithm performance using human resting state data. These recordings provide a generic, heterogeneous, and plentiful functional substrate against which to test different MEG recording and reconstruction approaches. We used a Hidden Markov model to spatio-temporally partition data into self-similar dynamic states. To test the anatomical precision that could be achieved, we then inverted these data onto libraries of systematically distorted subject-specific cortical meshes and compared the quality of the fit using cross validation and a Free energy metric. This revealed which inversion scheme was able to identify the least distorted (most accurate) anatomical models, and allowed us to quantify an upper bound on the mean anatomical distortion accordingly. We used two resting state datasets, one recorded with head-casts and one without. In the head-cast data, the Empirical Bayesian Beamformer (EBB) algorithm showed the best mean anatomical discrimination (3.7 mm) compared with Minimum Norm/LORETA (6.0 mm) and Multiple Sparse Priors (9.4 mm). This pattern was replicated in the second (conventional dataset) although with a marginally poorer (non-significant) prediction of the missing (cross-validated) data. Our findings suggest that the abundant resting state data now commonly available could be used to refine and validate MEG source reconstruction methods and/or recording paradigms.

## Highlights

• Resting state data provides an unbiased dataset for comparing MEG source estimates.
• We use a hidden Markov model to break the data into stationary segments.
• We reconstruct these data onto a range of distorted cortical surfaces.
• The sensitivity of any algorithm to spatial distortion is quantified in millimetres.

## 1 Introduction

Magnetoencephalography (MEG) detects electromagnetic fields at sensors outside the head. The challenge for the researcher is to infer the neuronal current distribution responsible for the observed data, despite a much higher number of possible sources than sensors. The general approach is to restrict the number of potential solutions through a priori assumptions, including the temporal relationship between sources (i.e. source co-variance) and/or the anatomical manifold that gives rise to this function (e.g. the cortical mesh). These assumptions are continually being refined and debated (Baillet, 2015; Baillet et al., 2001; Lin et al., 2006; Wipf and Nagarajan, 2009). Two recurring issues make it difficult for the community to come to a consensus on optimal source reconstruction methods - the first is the choice of test scenario, the second is the lack of ground truth.

Firstly, the choice of task, or simulation set-up used to compare source localisations will introduce a selection bias towards a specific temporal pattern predominant in certain cortical areas, which will suit some inversion assumptions but not others. Here we set out a framework which utilises diverse spatio-temporal patterns and minimizes selection bias by using a Hidden Markov model (Baker et al., 2014) to parcel endogenous resting-state data into collections of self-similar and quasi-stationary time segments. Resting state data have been shown to arise from dynamic spatio-temporal network state fluctuations occurring on the scale of 100–200 ms (Baker et al., 2014; Koenig et al., 2002; Wackermann et al., 1993; Woolrich et al., 2013) that include the rehearsal of the transient dynamic patterns observed during task performance (O'Neill et al., 2017). These networks predominate in all M/EEG recordings (even those which are task based) and are key to healthy brain function (Barttfeld et al., 2015; Kaiser et al., 2015; Lewis et al., 2009; Li et al., 2012; Peterson et al., 2014; Philippi et al., 2015; Reineberg et al., 2015; Sheline and Raichle, 2013; Tessitore et al., 2012; Venkataraman et al., 2012; Wu et al., 2014; Wurina et al., 2012). We rely on these iterant dynamics, rehearsing multiple task scenarios, to provide a varied and unbiased spatio-temporal repertoire of the source reconstruction problems one might expect from any dataset on which to then test our inversion schemes.

The second problem then, having identified an appropriate and representative real dataset (as opposed to simulated data), is the lack of access to the ground truth with which to compare recording/inversion techniques. Here we leverage new analytic techniques to quantify the sensitivity of MEG source inversion schemes by progressively deforming the anatomical models (Lopez et al., 2013; López et al., 2017; Stevenson et al., 2014). Specifically, we quantify how distortions in the MRI-extracted cortical manifold (mesh) affect our ability to predict or model the underlying current distribution (using cross validation error and Free energy). The technique assumes that the MEG sensor level data are due to current flow normal to the cortical surface but makes no assumptions about how this current should be distributed. The rationale is that the best MEG inversion scheme will be the most sensitive to subtle distortions of the cortical anatomy (as we know that MEG data derives from grey matter structure). This spatial distortion metric then provides a principled basis for comparing different a priori inversion assumptions (i.e. different algorithms) and recording techniques. We are aiming for a generic method to provide a benchmark to refine inversion (or recording) methods based on human electrophysiological data from multiple labs.

The paper proceeds as follows: we first parcel resting state datasets into brief epochs using a hidden Markov model (Baker et al., 2014). The epochs for the four dominant networks were then amalgamated into four network-specific datasets for each subject and taken forwards for inversion. These datasets were then inverted onto a library of subject-specific distorted meshes, for which we had control over the spatial detail available in the forward model. For each of these meshes, and for each inversion scheme, we quantified the model fit using cross validation and Free energy metrics. As expected, we found that the greater the distortion from the true cortical mesh, the poorer the model fit. We then used this spatial quantification to compare different inversion schemes (implemented as different co-variance prior assumptions). For these data, we found that the beamformer-based priors (EBB) were the most sensitive to small deviations from the true anatomy. In addition to distinguishing between algorithms, here we also tested whether we could use the same methods to distinguish between datasets collected with and without a head-cast (Meyer et al., 2017a; Troebinger et al., 2014a; 2014b), where the accuracy of forward model is more precisely known, and those collected without and found marginal (but not significant) differences.

## 2 Methods

### 2.1 MRI

Subjects underwent two MRI scans using a Siemens Tim Trio 3 T system (Erlangen, Germany). For the head-cast scan, the acquisition time was 3 min 42 s, in addition to 45 s for the localizer sequence. The sequence implemented was a radiofrequency (RF) and gradient spoiled T1 weighted 3D fast low angle shot (FLASH) sequence with image resolution 1 mm^3^ (1 mm slice thickness), field-of view set to 256, 256, and 192 mm along the phase (A–P), read (H–F), and partition (R–L; second 3D phase encoding direction) directions respectively. A single shot, high readout bandwidth (425 Hz/pixel) and minimum echo time (2.25 ms) was used. This sequence was optimized to preserve head and scalp structure (as opposed to brain structure). Repetition time was set to 7.96 ms and excitation flip angle set to 12° to ensure sufficient SNR. A partial Fourier (factor 6/8) acquisition was used in each phase-encoded direction to accelerate acquisition. For the anatomical scan later used to construct the cortical model, multiple parameter maps (MPM) were acquired to optimise spatial resolution of the brain image (to 0.8 mm). The sequence comprised three multi-echo 3D FLASH (fast low angle shot) scans, one RF transmit field map and one static magnetic (B0) field map scan (Weiskopf et al., 2013).

### 2.2 Head-cast construction

Scalp surfaces from the head-cast MRI data were extracted using SPM12 (http://www.fil.ion.ucl.ac.uk/spm/) by registering MRI images to a tissue probability map which classified voxels according to tissue makeup (e.g. skull, skin, grey matter etc.). The skin tissue probability map was transformed into a surface using the ‘isosurface’ function in MATLAB^®^ and then into standard template library format with the outlines of three fiducial coils digitally placed at conventional sites (left/right pre-auricular and nasion). Next, a positive head model was printed using a Zcorp 3D printer (600 × 540 dots per inch resolution) and this model placed inside a replica dewar-helmet with liquid resin poured between the two, resulting in a flexible, subject specific, foam head-cast with fiducial indentations in MRI-defined locations (Meyer et al., 2017a).

### 2.3 MEG recording

Resting state data was acquired from 12 healthy subjects using head-casts (age: 26.6 ± 3.5 yrs (mean + sd)) and 12 other healthy subjects without head-casts (age: 25.2 ± 6.6 yrs). All subjects were right handed, had normal or corrected-to-normal vision, and had no history of neurological or psychiatric disease. Informed written consent was given by all subjects and recordings were carried out after obtaining ethical approval from the University College London ethics committee (ref. number 3090/001).

All subjects underwent a 10 min resting state scan with eyes kept open and instructed to fixate on a central cross on a screen, using a CTF 275 Omega MEG system. The head was localised using the three head-cast-embedded fiducials (head-cast subjects) or fiducials placed on the nasion and left and right pre-auricular points (non-head-cast subjects). Average range of absolute head movement within the 10 min resting state recording was 0.26 ± 0.06, 0.24 ± 0.05, 1.1 ± 0.54 mm (X,Y,Z directions; ± SEM) for head-cast and 3.2 ± 0.5, 3.0 ± 0.5, 3.3 ± 0.2 (X,Y,Z directions; ± SEM) for non-head-cast data. The data were sampled at a rate of 1200 Hz, imported into SPM12 and filtered (4th order butterworth bandpass filter: 1–90 Hz, 4th order butterworth bandstop filter 48–52 Hz) and downsampled to 250 Hz.

**Fig. 1:**
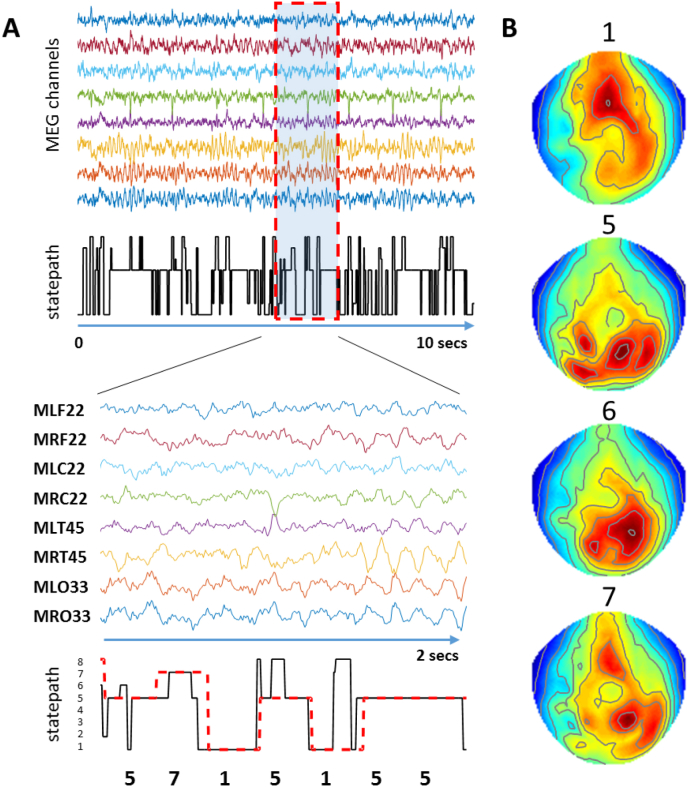
**MEG data and Hidden Markov Model network allocation. A.** Top panels show a selection of MEG sensor time series for head-cast subject 1. Middle panel demonstrates the statepath as determined by the HMM, showing rapid transitions between different states. Timecourses recorded from a random subset of sensors is shown. Bottom panel shows a short (2s) period of data (MEG channel labels e.g. MLF22 shown), expanded with the corresponding section of the statepath (black line). Overlying the statepath trace (black) is the modal statepath for each 200 ms period (dash red line) and this modal statepath trace was used to sort each 200 ms data epoch into 1 of 8 states according to which state was most frequent during that period. These epochs of data for each of these states were aggregated together to form 8 new datasets. **B.** The statepath traces for the four most dominant states here (1, 5, 6, 7) are shown correlated against the time series amplitudes in all sensors to derive a map of activity (red, greater positive correlation, blue, less positive correlation) associated with each particular state. Data are shown in sensor space in order to check that differential network parcellation had indeed occurred according to the statepath detection method and was not artefactual (e.g. that the topography resembles that expected for an eye blink). In the example shown here – state 5 is the most common state – corresponding to the posterior alpha rhythm.

Traditional inverse problem solutions are based on the assumption that the data are stationary during the period of inversion. However, resting state data contains rapid dynamics that do not accord well with this assumption (Woolrich et al., 2013). Therefore, in order to improve stationarity within a given epoch, we parcellated the data into self-similar periods that capture the resting state network transitions (100–200 ms) using a Hidden Markov Model (HMM) that could identify the rapid formation and dissolution of recurring resting state networks (Baker et al., 2014). With this, a ‘statepath’ was estimated for each 10 min resting state block, which tracks the fine spatiotemporal dynamics and allocates each point in time to one of eight dominant network states (Baker et al., 2014). For this statepath determination, a copy of each subject's sensor level data was dimensionally reduced using principle component analysis (PCA) to derive 40 components of unit variance and mean (Woolrich et al., 2013). With these data, an 8 state Hidden Markov model (HMM; www.fmrib.ox.ac.uk/∼woolrich/HMMtoolbox) was then applied to derive the most probable state at all points in time (the statepath) (Fig. 1A) (Baker et al., 2014). The continuous 600s of data was then epoched into 200 ms blocks/epochs (the average scale of individual state transitions (Baker et al., 2014) and each 200 ms data epoch allocated to a new dataset according to the state that was most dominant during that time period. This resulted in 8 new datasets each with a collection of epochs that corresponded to a distinct network state as identified by the HMM. The four most dominant states for each individual subject (as measured by most time spent in that state) was taken forward for further analysis and spatial estimates averaged across those four inversions for each subject. As the HMM was performed on individual, rather than group concatenated data, state numbers did not directly correspond across subjects. This however permits superior partitioning within subjects since it allows the model to optimally fit states to the individual subjects, rather than fitting individual data to group states that are (or are not) common across all subjects. This resulted in 815 ± 56.9 data segments per partitioned dataset - equivalent to 163s of continuous data. Since the HMM selected periods of self-similarity within the resting state, partial correlation maps were examined for all subjects to check that segregation into separate networks had occurred and this segregation did not relate to eye blinks, muscle artefacts or cardiac interference (Fig. 1B).

### 2.4 Subject-specific cortical mesh libraries

**Fig. 2:**
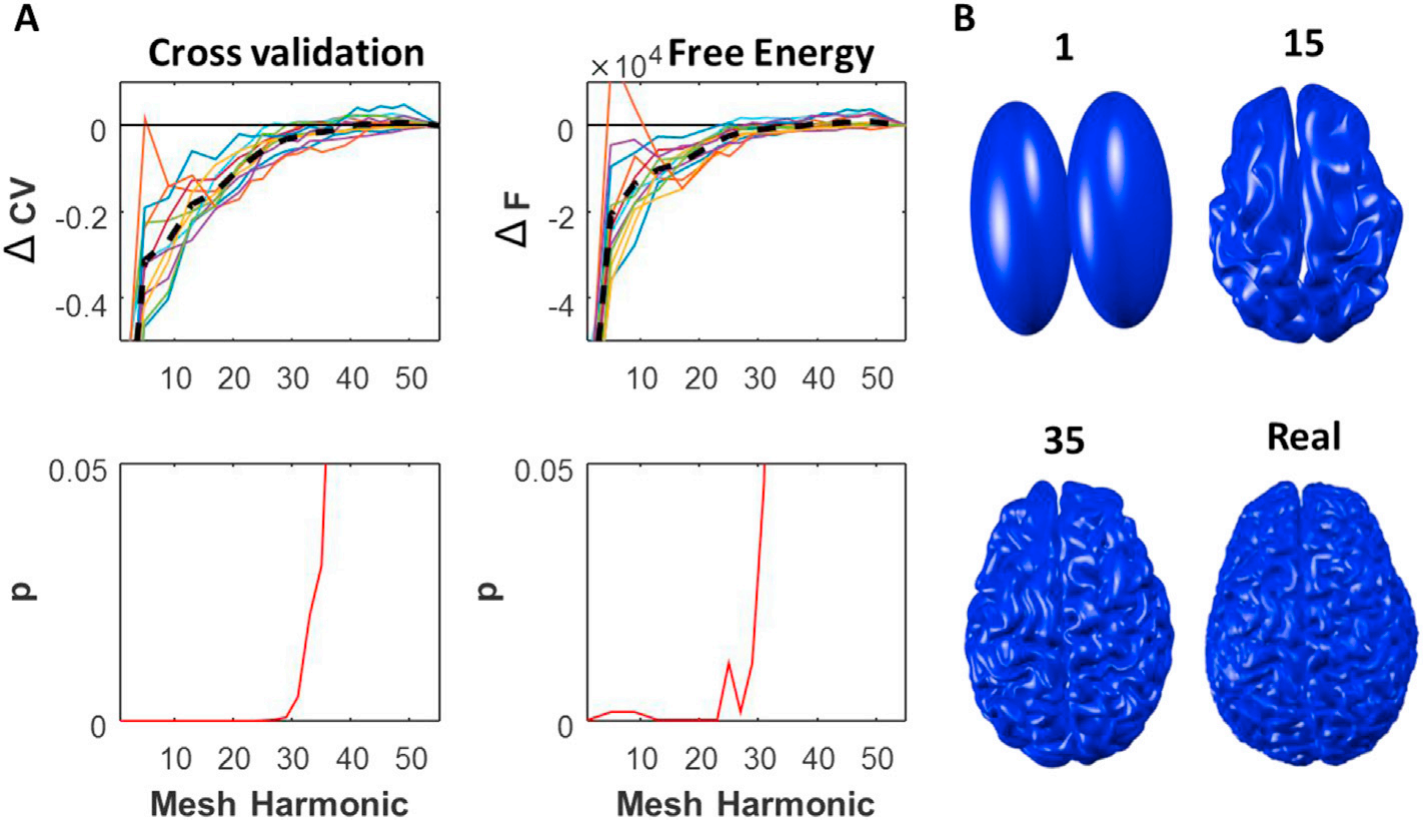
**Relative Cross Validation and Free energy results for a library of different meshes for head-cast resting data using EBB inversion. A.** Increasing cross validation data explained (ΔCV) and relative Free energy ΔF with improving spatial resolution of harmonic meshes (from left to right). Top plots show individual subject ΔCV (left) and ΔF (right) values with superimposed mean value (dashed black line). Lower panels show the group level statistical significance using *t*-test (ΔCV) and Bayesian model comparison (ΔF) for cross validation and Free energy respectively. **B.** Example selection of meshes from subject 1 showing different levels of distortion, from smooth ovoid surfaces (WFS mesh 1) up to the real mesh.

To extract the cortical pial mesh surface, we used Freesurfer software optimized for MPM scans by using PD and T1 volumes as inputs. A detailed description of these methods of cortical surface reconstruction and optimization can be found in earlier work (Carey et al., 2017; Lutti et al., 2014). Then, for each individual subject, the pial cortical mesh was taken and deformed using a 3D weighted Fourier analysis that effectively decomposes the original 3D mesh structure into spatial harmonic components (Chung et al., 2007). These are then sequentially combined to form a set of meshes of progressively increasing spatial detail (Fig. 2B) (Stevenson et al., 2014). These meshes are called the Weighted Fourier Series (WFS) and result in a library of meshes for each subject with different levels of spatial detail, from a completely smooth pair of ovoid surfaces (mesh 1) up to a mesh which is very similar to that extracted from the MRI of the subject's brain (mesh 50) (see Fig. 2B). The WFS can be expressed as follows:

$$F_{\sigma }^{k}\left[f\right]\left(\omega \right)=\sum_{l=0}^{L}\sum_{m=-1}^{l}e^{-l\left(l+1\right)}f_{lm}S_{lm}\left(w\right)$$

where σ is the bandwidth of the smoothing kernel (set at 0.0001), *L* is the harmonic order of the surface, *S*_*lm*_ is the spherical harmonic of degree l and order m, and the Fourier coefficients are given by <*f*_*lm*_=*f, S*_*lm*_>, where f is determined by solving a system of linear equations (Chung et al., 2007). All meshes, including the true mesh, were downsampled by a factor of 10 in Freesurfer, to ∼33,000 vertices per mesh to aid computational efficiency. This resulted in a mean vertex distance to nearest neighbour of 1.7 mm (mean within-subject 5 & 95 percentiles 0.8–2.6 mm).

### 2.5 Source reconstruction

We used SPM12 which implements multiple different inversion schemes within a common empirical Bayesian pipeline, whereby all processing steps, besides the choice of prior source co-variance matrix are held constant (Friston et al., 2008; López et al., 2014). The choices of prior source covariance matrices used embody four popular inversion algorithms: The traditional unweighted Minimum norm (MMN) in which the source covariance is a diagonal (IID) matrix (Hämäläinen and Ilmoniemi, 1994). An (unweighted) LORETA-like solution (Pascual-Marqui, 2002) based on a surface (rather than volumetric) smoothing using Green's function (Harrison et al., 2007) (LOR). An Empirical Bayesian Beamformer (EBB) inversion (Belardinelli et al., 2012) in which a direct estimate of prior source co-variance is made based on the sensor-level data (and zero regularization). The main difference between the EBB scheme and the classic linearly constrained minimum variance (LCMV) (Van Veen et al., 1997) scheme is that the EBB attempts to fit the measured data (by optimizing model evidence). This has the advantage that EBB attempts to explain the measured data and can therefore provide a direct model evidence metric (which can then be compared to other linear inversion algorithms); but the disadvantage is that if there is external noise in the measured data the Bayesian scheme will still attempt to fit it (unless appropriately modelled) whereas the LCMV (spatially filtered) solution will be unaffected. The multiple sparse priors algorithm (MSP) (Friston et al., 2008) uses an optimized search to select mixtures of source covariance matrices each describing the activity of separate patches of cortex. For each of the different algorithms, the source level estimates were weighted against a diagonal sensor noise covariance matrix (effectively regularizing the solution) within the same empirical Bayes framework. For more details on each of these methods and the empirical Bayesian optimization please see (Belardinelli et al., 2012; Friston et al., 2008; López et al., 2014).

The HMM parcellated datasets originally contained 272 channels and 50 time samples (3 channels were damaged and excluded from the data). As part of the cross validation procedure, we excluded 10% of these channels from the inversion stage, leaving 245 channels of data. This procedure left 27 unused channels out of the inversion, the data from which were then predicted based on the source estimates and compared to the real sensor data. For each of the ten cross-validation folds (each time a random 10% of channels were selected to be left out), the remaining (245 channels of) data were temporally decomposed into 16 orthogonal temporal modes and inverted onto each surface from the library of individualised and distorted cortical meshes (WFS) for each subject. Each inversion provided 10 cross-validation scores (the prediction of the hidden channels) which were then converted to a percentage of source data explained (more positive = better model fit) and averaged across the 10 folds and across the four state datasets inverted per subject.

Both cross validation and Free energy provide metrics which can be used to directly compare different models of the same data, with different independent methods of preventing over-fitting (Bonaiuto et al., 2018; Henson et al., 2009). Whilst the Free energy provides a useful relative model fit metric for any given dataset, the absolute value is data dependent and therefore cannot be used to compare between datasets. In contrast, the cross validation gives a meaningful quantification of the total amount of data explained and can be used to compare across the different groups (e.g. head-cast versus non head-cast). After converting cross validation error to the percentage of total data explained by the model, both the CV % data explained and the Free energy increase with improving model fit.

Here we performed these inversions using our subject specific libraries of anatomically degraded meshes from the WFS with different levels of distortion and compared them to an inversion performed using the real mesh for each subject. We constrained the source estimates to lie on the cortical surface and normal to that surface (Phillips et al., 2002). In order to facilitate comparisons of how the anatomical distortion affected the inversions for different subjects with different baseline measures of model fit to their real brain meshes, we normalised the cross validation percentage data explained and Free energy metrics by subtracting the values for the real mesh to derive a relative measure (ΔCV and ΔF). As such, a worse fit gives a negative value of ΔCV (and ΔF) and we would predict that as the anatomical complexity of the mesh increases (mesh is less distorted) and approaches that of the real mesh – the quality of the model should improve and the ΔCV and ΔF values should approach zero. Across our group of subjects, we determined the level of distortion (harmonic) at which this first becomes statistically distinguishable from the real mesh using a *t*-test of ΔCV values for each harmonic compared to zero. Similarly for ΔF, we use Bayesian model comparison to find out how much distortion is needed to make two meshes distinct. For example, using algorithm A we may find that inversions are not much worse (ΔCV and ΔF not significantly different from zero at the group level) using very distorted meshes all the way down to quite a smooth mesh harmonic (e.g. <10) i.e. the inversion algorithm is very insensitive to changes in the mesh shape. Alternatively, using algorithm B, we find that even with very subtle distortions of the mesh, that the inversion fit deteriorates substantially, so down at just harmonic 40 (which looks to the naked eye to be very similar to real mesh) the ΔCV and ΔF values are significantly below zero at the group level. This point (10 and 40 in this example), labelled the highest distinguishable harmonic (HDH), identifies the minimum amount of mesh distortion that can be reliably distinguished from the real mesh by inversion and can be converted into a conventional spatial metric (mms) by comparing vertex distances. The three dimensional Euclidean distance between every vertex from the HDH and the corresponding vertex on the true mesh was therefore calculated to give a distribution of 33,000 distances between the real mesh and the first mesh that can be distinguished from it statistically by inversion. We then took the upper, 95th percentile as an estimate of the distance between the two meshes. In addition to using the 95th percentile rather than the mean, this method is more conservative than that previously employed (Stevenson et al., 2014), as this directly matches corresponding vertices and therefore reduces the underestimation that could result if, following harmonic distortion, a vertex now lies closer to a non-corresponding other vertex. This distance therefore represents an upper bound on the spatial discriminability of both head-cast and non-head-cast resting state data.

### 2.6 Control analyses

In order to verify our findings, we performed a number of control analyses. Firstly, we used the same data but destroyed its correspondence with the MEG sensor locations by randomly shuffling the MEG channels labels and repeated the analysis above 10 times for each subject and averaged over the ΔCV for these different shuffled dataset inversions. It is worth noting that sensor shuffling also removes all the (real) spatial correlations between sensors, however since they are not independent, they are not perfectly exchangeable and thus we also performed a secondary control analysis. For this, we used the correct sensor labels but instead degraded our data by introducing different amounts of uncorrelated scaled white noise at the sensor level to change the signal-to-noise ratio of the sensor level data (5 dB to - 20 dB) (Troebinger et al., 2014a). In both cases (sensor shuffling and noise addition), one would expect the ability to discriminate the true generative model from distorted ones to decrease.

## 3 Results

### 3.1 Anatomical cortical model

In order to determine the sensitivity of the inversion to the level of detail in the underlying anatomical mesh model we calculated the ΔCV and ΔF for each subject in the head-cast dataset across their subject-specific library of distorted meshes (Fig. 2). This showed increasing cross validation sensor data explained (ΔCV; reduced error) and increasing Free energy (ΔF) for all subjects. Statistical group level testing revealed that meshes lower (more deformed) than the 35 harmonic could be distinguished from the real mesh by ΔCV (HDH 35; t_11_ = −2.49, p = 0.03) and lower than 31 by ΔF (BMC, exceedance p = 0.046; Fig. 2). Having confirmed our results using the two metrics – we proceeded with the ΔCV metric which can be compared across subjects and recording methods.

### 3.2 Inversion algorithms/source covariance priors

**Fig. 3:**
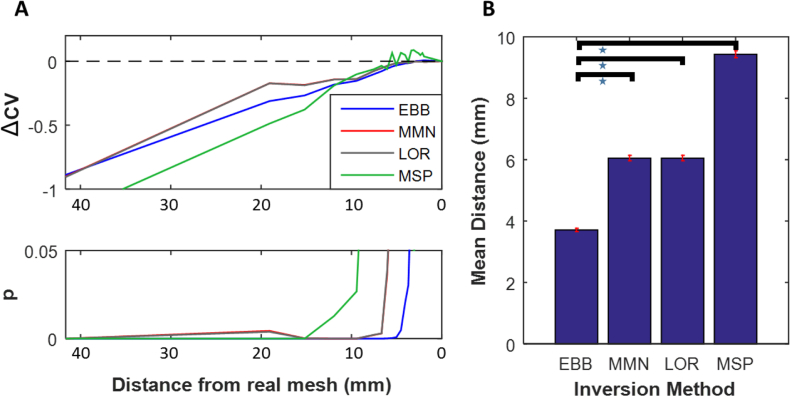
**Effect of different inversion schemes on head-cast resting state resolution. A.** Relative cross validation data explained (ΔCV) for head-cast MEG dataset (averaged across 12 subjects), shown for 4 different inversion types (EBB, MMN, LOR & MSP) according to mean distance of the distorted mesh (x axis) from the real mesh. Lower panel shows the group level statistical significance by t-testing (note MMN (red) and LOR (grey) have very similar values and therefore lines are closely overlapping). **B.** Mean distance of vertices from highest significant mesh identified in the left hand panel and the real mesh. Error bars show SEM of this distance across subjects using their individualised meshes and group level HDH.

The effect of source co-variance prior assumptions was then assessed by repeating the process for three other commonly implemented inversion algorithms (MMN, LOR, MSP) on our head-cast dataset. These showed lower anatomical mesh discriminability for all alternative algorithms with an HDH for MMN of 25 (t_11_ = −1.80 -, p = 0.036), an HDH of 25 (t_11_ = −1.79; p = 0.039) for LOR and an HDH of 17 for MSP (t_11_ = −2.55; p = 0.027) (Fig. 3A).

The mean distance between vertices on these meshes and corresponding vertices on the real (MRI-extracted) mesh was then calculated for each subject. These were then averaged to give a spatial measure of anatomical discriminability (Fig. 3A), under the different prior covariance assumptions as implemented in the different inversion algorithms. This ranged from 3.7 mm for EBB to 6.0 mm for MNN/LOR and 9.4 mm for MSP (Fig. 3B). Directly comparing the amount of data explained through cross validation across inversion conditions (using the real mesh) demonstrated a significant difference between EBB and the other 3 algorithms (EBB/MMN; paired *t*-test – t_11_ = 7.6,p < 0.001; EBB/LOR; paired *t*-test – t_11_ = 7.6,p < 0.001; EBB/MSP; paired *t*-test – t_11_ = 14.4,p < 0.001).

A 2-factor within-subject ANOVA of % cross validation data explained with factors – inversion type (EBB, MMN, MSP) and mesh smoothness (harmonic 1 and harmonic 50) showed a significant interaction between inversion type and mesh harmonic level (F_2_ = 29.9, p < 0.0001). Post hoc examination showed that this was driven by a stronger effect of mesh distortion on the MSP inversion algorithm than EBB or MMN.

### 3.3 Head-cast versus conventional MEG

**Fig. 4:**
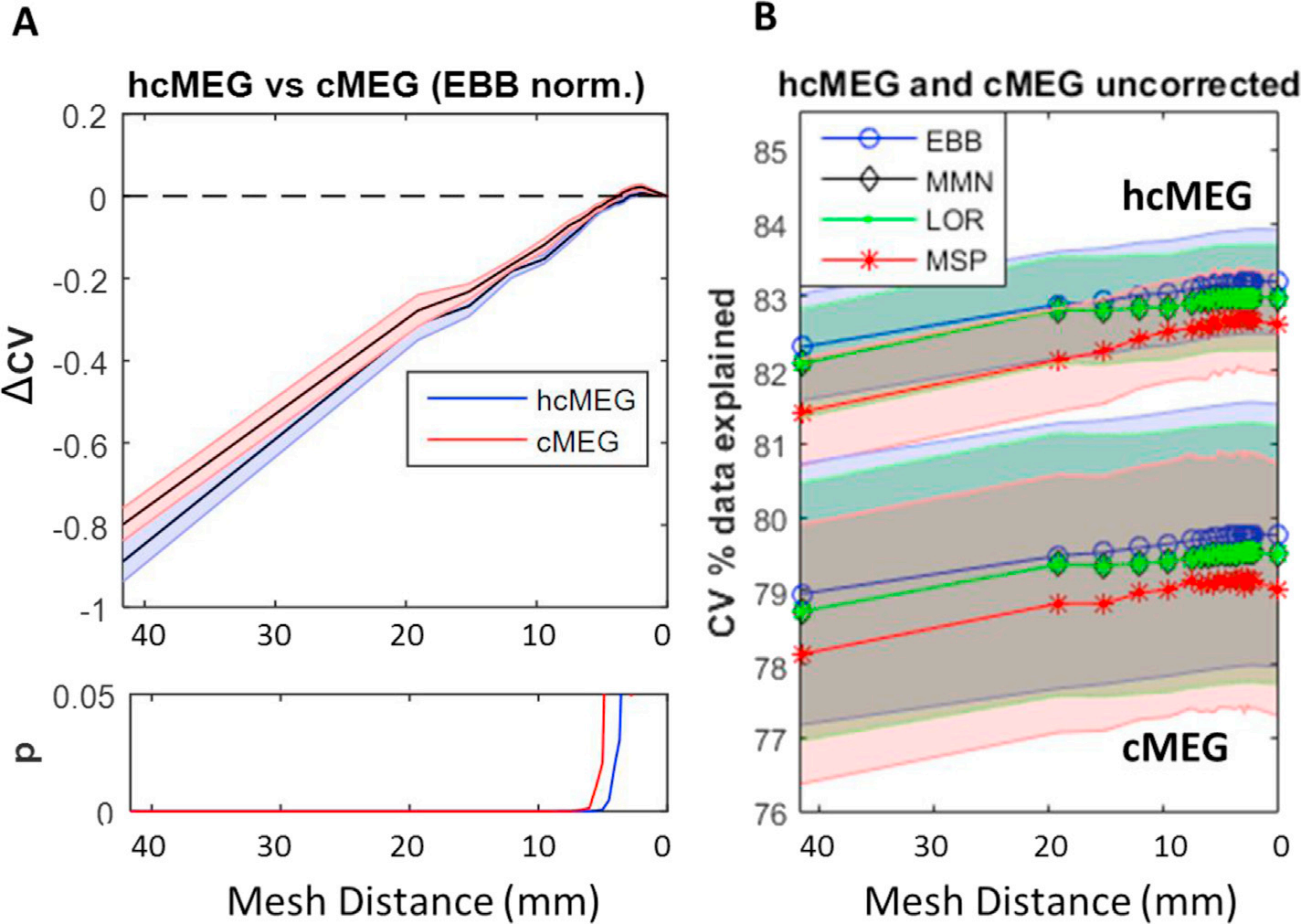
**Comparison of head-cast versus conventional MEG cross validation accuracy. A.** Comparison of ΔCV for different recording methodologies with head-cast (hcMEG) data showing higher discrimination (3.7 mm) than conventional MEG (cMEG) data (5.0 mm). **B.** Absolute cross validation data explained is also higher in the head-cast versus the conventional MEG. Note that this holds for all inversion types and for all levels of mesh distortion, but was not significantly different (F = 3.06, p = 0.09).

The EBB algorithm was therefore taken forwards for a comparison of head-cast versus non-head-cast datasets (Fig. 4). We found that the HDH was higher for the head-cast recorded dataset at 35 (3.7 mm; t_11_ = −2.49, p = 0.03) than for the non-head-cast related dataset at 29 (5.0 mm, t_11_ = −2.70, p = 0.021). Furthermore, the absolute (non-normalised) amount of data explained through cross validation (CV) was higher in the head-cast (83.2 ± 0.71%) than in the conventional recordings (79.7 ± 1.79%) although this difference was not significant (mixed ANOVA; main effect of hcMEG versus cMEG, F = 3.06; p = 0.09).

### 3.4 Control analyses

**Fig. 5:**
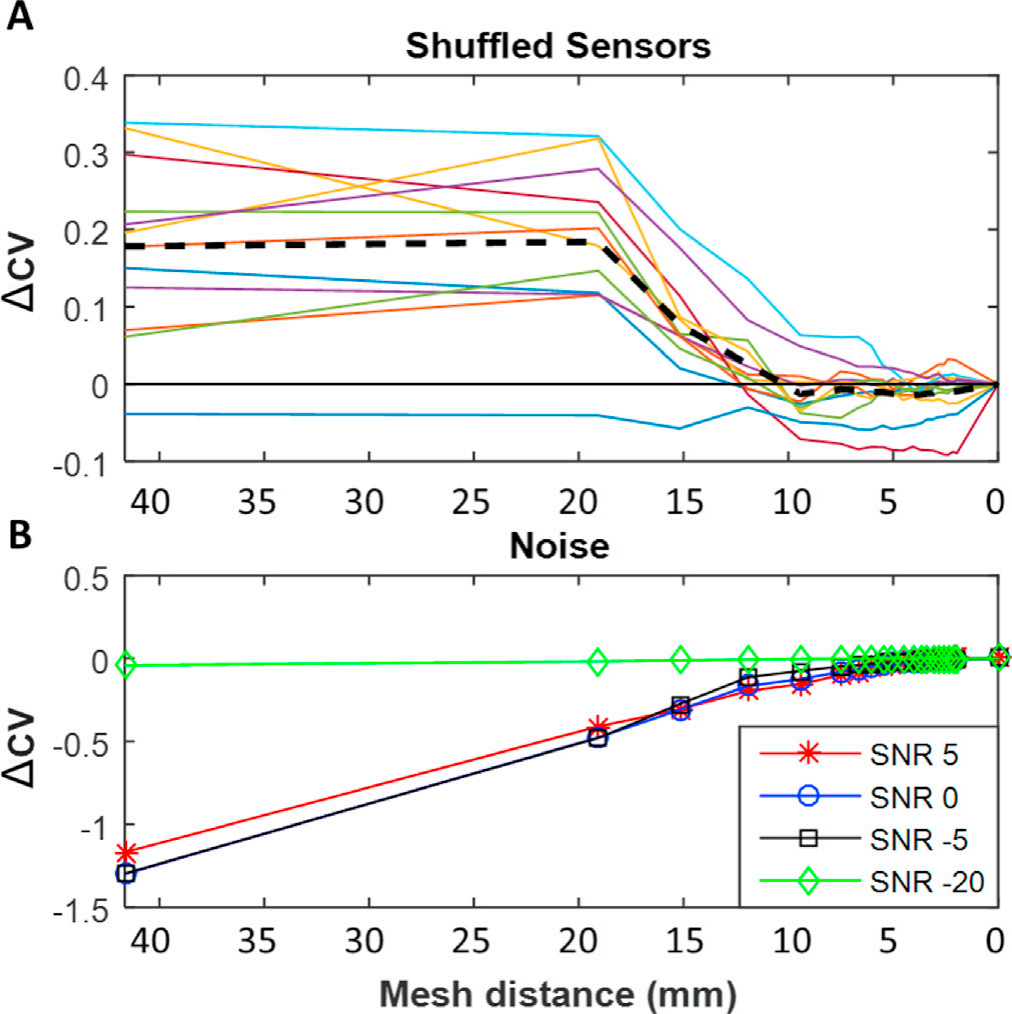
**Effects of shuffling sensors (A) and replacing data with varying levels of Gaussian White noise (B). A.** The relationship between the percentage of data explained through cross validation (ΔCV) of inversion (normalised to the real mesh) and increasing harmonic mesh after random shuffling of sensor labels. Note the decreasing cross validation fit with increasing mesh harmonic (increasing mesh detail) with the dashed line showing the average across all subjects. **B.** The relationship between (mean of all 12 subjects) ΔCV of inversion and increasing harmonic mesh after addition of Gaussian white noise across a range of noise additions (from SNR 5 to SNR -20 in decibels (dB)).

Finally, we checked our analyses by repeating our inversions (Head-cast dataset, EBB algorithm) but after degrading the consistent relationship between our sensor positions and sources by shuffling the sensor labels. As expected, this resulted in a breakdown of the previously shown relationship between cross validation and Free energy with mesh distortion. Notably we found that lower harmonics (smoother) meshes now showed higher ΔCV (Fig. 5A) (smoother surfaces superior when sensors shuffled). Thereafter we degraded our data (without sensor shuffling) by the addition of varying levels of Gaussian white noise (5 to −20 dB) and again repeated our analysis (Fig. 5B). This demonstrated that with increasing levels of noise (decreasing SNR), the curve describing the relationship between cross validation and harmonic mesh function flattened and the crossing point (HDH) reduced, indicating that the inversion was no longer able to statistically distinguish the more complex meshes from the real mesh, as would be expected if the data are primarily noise.

## 4 Discussion

Resting state data is dynamic, emulates task induced network changes, is predictive of behaviour and is simple to acquire in both control and patient populations (Baker et al., 2014; Larson-Prior et al., 2013; Niso et al., 2016; O'Neill et al., 2017; Philippi et al., 2015; Taylor et al., 2017). Here we show that it can provide a ready substrate for principled testing of MEG recording methods and inversion assumptions including anatomical forward modelling and functional (co-variance) priors.

We showed that in moving the cortical surface from a heavily distorted version to the true anatomy, a significant and monotonic improvement occurred in model fit. This improvement saturated for some inversion schemes before others, with the beamformer based algorithms (closely followed by Minimum norm) continuing to improve up until the cortical surface deviated by, on average, <4 mm from the ground-truth. Critically, rather than compare methods through simulation or a limited task set (with ground truth from another modality), we have presented a method to optimise MEG forward and inverse models whilst minimizing selection bias and based on a plentiful supply of non-invasive human data. The HMM constrained the source estimates to select out self-similar and stationary epochs – under the assumption that this would also lead to a clearer picture of the underlying source distributions (essentially segmenting multiple overlapping source distributions into different temporal parcels and therefore simplifying the source reconstruction problem). We tested whether this step was justified by comparing the resolution estimates from the HMM parcelled data with arbitrary mixtures of parcels (supplementary Fig. S1). We found that the HMM approach demonstrated a significantly higher resolution in each subject compared to the non-HMM dataset (t11 = 3.18, p < 0.009). An alternative scheme, avoiding both resting state and the HMM, would be to define stationary periods of time around a common battery of tasks/stimuli to be used (but first agreed upon) universally; however, the concern would be that the ensuing cortical responses might still only occupy a small portion of the possible spatio-temporal solution space.

We then compared algorithms in two ways: by the model fit (or amount of data predicted), and by the sensitivity of each algorithm to the true anatomy. These two tests need not necessarily have been in accord. For example, had we used a bunny-shaped blancmange mould instead of a cortical surface, we would still have been able to rank the algorithms based on the amount of data predicted; but we would not have expected any monotonic improvement as features were added to the bunny. A related control analysis (Fig. 5) is that when we used the same data but with shuffled lead-fields (destroying the link between the sensors and the anatomy) the amount of data we are able to predict actually decreases as the cortical model approaches the truth. It is therefore striking that the models that benefitted most from the true cortical manifold were also those that predicted the most data. This adds anatomical validity, confirming that the data being described is indeed generated by pyramidal cell populations normal to the cortical surface. Furthermore however, it also allows us to quantify algorithm performance in millimetres without being restricted to or dependent on using a particular processing framework (e.g. SPM) or cost function (e.g Free energy or cross validation) (Stevenson et al., 2014).

Across anatomy (Fig. 2) and inversion assumptions (Fig. 3), the parametric (Free energy from empirical Bayes) and non-parametric (cross-validation) metrics of model fit were in accordance. This helps build confidence in the parametric Free energy metric which is considerably faster, makes use of all the available data, and has a direct probabilistic interpretation (i.e. how much more likely is one model than another?). The Bayesian formalism is however predicated on comparing how different models explain the same data; the use of cross-validation, which provides an absolute quantitative measure of data predicted, also allowed us to compare between different datasets (head-cast versus non-head-cast). We confirm here earlier reports that within-session head movements are reduced for head-cast versus non-head-cast MEG (Bonaiuto et al., 2017; Liuzzi et al., 2017; Meyer et al., 2017a; 2017b). We were surprised that the head-cast and non-head-cast data were statistically indistinguishable in terms of spatial resolution (mixed ANOVA; main effect of hcMEG versus cMEG, F = 3.06; p = 0.09). However, we believe that the comparison was particularly conservative in that the non-headcast group showed very little average head movement (3 mm), comparable to the average mesh vertex spacing (1.7 mm). Another limitation could be that the models we are using do not sufficiently capture the physics or physiology of the generators of the measured magnetic fields; and that the resolution is constrained by the models and not the recording. This could include for example, un-modelled noise sources such as the heartbeat, eye-blinks and other sources of noise. Although the HMM states we used were visually inspected to avoid common artefacts such as eye-blinks, it is possible that some of the modelling deficiencies come from failure to explain data that does not arise from the cortex (e.g. heart-beats). Finally, the head-cast and non-head-cast cohorts did not overlap and a more sensitive analysis would have been to perform a within-subject comparison.

Here we used individualised meshes with current flow constrained to be normal to the cortical surface. These normal constraints, based on the MRI surface extraction, will themselves have some noise (which will be attenuated by smoothing in the lower harmonic orders) and could be one of the limiting factors in our spatial resolution estimates. One further consideration was that our estimates of resolution might have been confounded by absolute levels of signal power. We found no evidence for this in any frequency band (supplementary Fig. S2). We did, however, find that our subject group was divided into subjects showing a dominant theta/delta peak and those showing a peak in the alpha range (supplementary Fig. S2). We found that consistent with previous (MEG within-subject) findings (Stevenson et al., 2014), we achieved a greater sensitivity to spatial distortion in the subjects with a peak in a higher frequency range (p < 0.002). This is consistent with invasive observations that higher frequency oscillations tend to have smaller spatial coherence domains (Leopold et al., 2003). We should note however that the theta cycle is close to our chosen window length (200 ms) and this could be another reason for the lower spatial resolution estimates in those subjects dominated by lower frequencies.

We found that the Multiple Sparse Priors algorithm had the least dependence on the true anatomy and also explained the least data. We should note however that the MSP-based analyses implemented here were generic and constructed from a limited set of 512 patches (or priors) placed at approximately evenly spaced vertices. The MSP algorithm, although perhaps the most elegant and comprehensive method we tested, is also computationally disadvantaged by the need to search over a large space of possible patch/prior combinations and the inherent pitfalls of local extrema in this optimization. A more robust way to implement this algorithm would have been to select the best model from many random patch choices (Troebinger et al., 2014b). Moreover, the relationship between sparsity assumed by the algorithm, and resting state networks used here for analysis, is beyond the scope of this study. We should note that the implementations of all the algorithms we tested were generic and not individually optimized. For example, had we used an optimized MNE framework (Gramfort et al., 2014) then no doubt its relative sensitivity would have improved. Here we focus on developing the benchmark and showing its application, rather than an exhaustive comparison of algorithms.

This study was analysed using broadband (1–90 Hz) resting state data. Therefore, whether a similar level of spatial discriminability at the mm scale can be demonstrated when more selective data is used (e.g. frequency filtered or spatially restricted) remains to be shown. For example, future work might test different frequency bands (eg <30 Hz, >30 Hz) against different anatomy for example infra/supra granular cortical surfaces (Arnal and Giraud, 2012; Bastos et al., 2012; Bonaiuto et al., 2018, 2017).

Overall, we have here demonstrated and validated a robust and unbiased method for the comparison of inversion and MEG recording methods that can in future be used to evaluate and optimise future techniques.

## Appendix A Supplementary data

RESTING_STATE_PAPER[19-21] RESTING_STATE_PAPER[19-21]
