# Supplementary material for: Quantifying the performance of MEG source reconstruction using resting state data
Source: Neuroimage. 2018 Nov 1;181:453–60. doi: 10.1016/j.neuroimage.2018.07.030 (PMC6150947; doi:10.1016/j.neuroimage.2018.07.030)
Supplement: RESTING_STATE_PAPER[19-21] [file mmc1.docx]

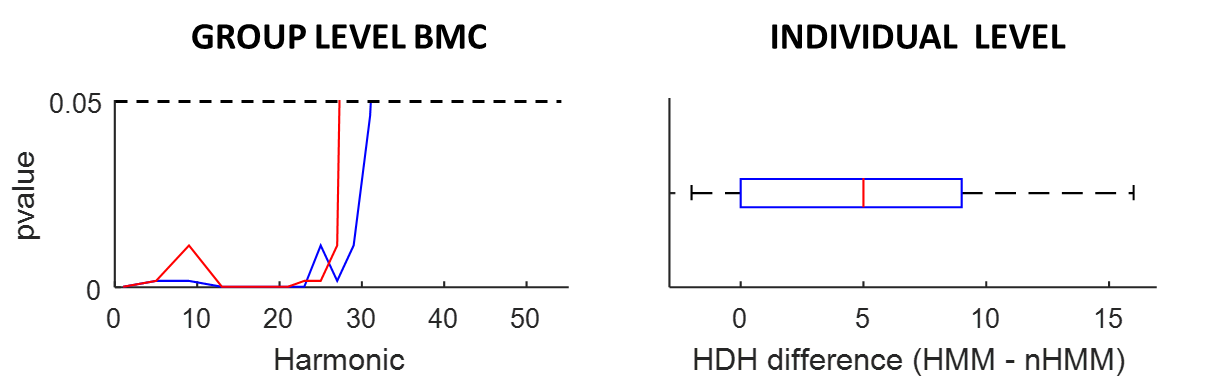


**Supplementary Figure S1. Demonstration of the effect of using HMM parcellated data versus arbitrary, non-selected, epochs on spatial inversion resolution at the group and individual subject levels.** The left panel shows the group level statistics using Bayesian Model Comparison (BMC) for HMM (blue) versus nHMM datasets (red), compared to the real mesh. Note that the HMM data are distinguishable from the real mesh at a higher harmonic (31) than the nHMM (27). The right panel shows the difference for each subject for the highest harmonic that can be distinguished at the individual level (Free energy difference between the distorted brain and real mesh of less than 3) which is consistently higher for the HMM versus the nHMM datasets (t11=3.18, p< 0.009).


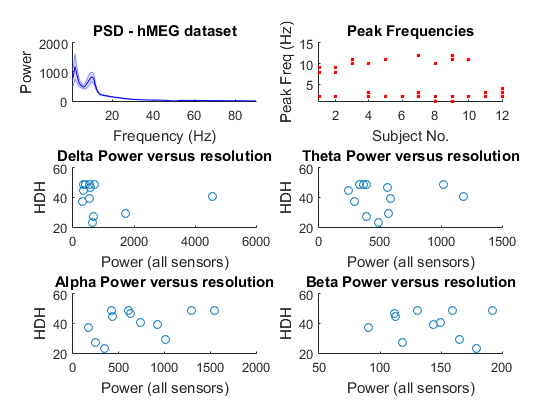

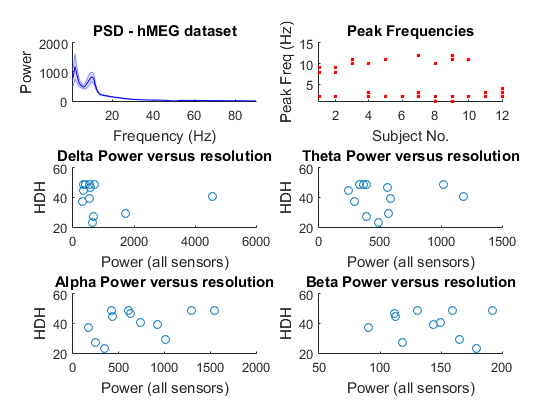


**A**

**B**

**C**

**Supplementary Figure S2. Spectral features of sensor level data.** Panel A shows the Power Spectral Density (PSD) averaged over all sensors/subjects/networks. We found that the majority of the signal power within and between subjects were focused in the lower frequency bands. Panel B shows the range of peak frequencies within and across subjects (up to 4 markers per subject according to HMM network, but some networks have the same peak frequency and are therefore overlapping). Over subjects the peak frequency tended to be either in the alpha (8-12Hz) or delta (1-4Hz) range. Panel C - when testing whether the absolute power in any frequency band could predict the resolution metric that we derived (panels C-F), we found no significant correlation in any band (Delta p=0.69; Theta p=0.83; Alpha p=0.20; Beta p=0.72).

**Supplementary Figure S3. Scatter plot of subject peak sensor level frequency against highest discriminable harmonic for that subject.** Subjects with a spectral peak in the low frequency (<5Hz) range at the sensor level were less sensitive to small mesh distortions (the HDH metric) than those with sensor level peak in the higher frequency range. Individual subjects are shown with blue circles alongside the best fit regression line (orange).
